# Bipolar Disorder Comorbid with Arnold-Chiari Malformation: Case Report

**DOI:** 10.1192/j.eurpsy.2022.1059

**Published:** 2022-09-01

**Authors:** Z. Yalçın, O. Sahmelikoglu Onur, N. Karamustafalioglu

**Affiliations:** Bakirkoy Research & Training Hospital for Psychiatry, Neurology and Neurosurgery, Psychiatry, Istanbul, Turkey

**Keywords:** mood disorder, bipolar disorder, Arnold-Chiari Malformation

## Abstract

**Introduction:**

Arnold Chiari malformation (ACM), a condition in which a portion of the brain pushes through the opening at the base of the skull, can cause headaches, dizziness, difficulty swallowing, muscle weakness and balance problems. The prevalence in the general population has been estimated at slightly less than 1/1000. The majority of these cases are asymptomatic. Chiari malformations are often detected coincidently among patients who have undergone diagnostic imaging for unrelated reasons. Several cases of psychiatric illness comorbid with ACM type 1 (ACM1) are reported in the literature.

**Objectives:**

Here we reported a patient with bipolar affective disorder, manic episode with a history of depressive episodes for 2 years comorbid with ACM1.

**Methods:**

A 39 year-old-woman,with the history of panic disorder and obsessive compulsive disorder comorbid with depression have been using sertraline 50 mg/day for a year, admitted for decreased need of sleep, grandiosity, increased libido,risky behaviours, rapid speech and agitation. The patient met DSM 5 criteria for a manic episode and was hospitalized. She had a positive history of depression in her family. Her lab work up was unremarkable; including negative urine toxicology. MRI scans, for exclusion of organicity, demonstrated ACM1. Her treatment was started with a regimen of haloperidol 20 mg/day, biperiden 10 mg/day. The treatment was switched to olanzapine 20mg/day upon detection of rigidity. Lithium was added as 900mg/day. Neurosurgery, outpatient control was recommended by neurosurgery.

**Results:**

The patient’s symptoms gradually improved within one week with attainment of euthymic mood.

**Conclusions:**

This case might show that ACM1 could cause abnormal functioning of brain circuits promoting psychiatric symptoms.

**Disclosure:**

No significant relationships.

